# Skeletal muscle atrophy in heart failure with diabetes: from molecular mechanisms to clinical evidence

**DOI:** 10.1002/ehf2.13121

**Published:** 2020-11-22

**Authors:** Nathanael Wood, Sam Straw, Mattia Scalabrin, Lee D. Roberts, Klaus K. Witte, Thomas Scott Bowen

**Affiliations:** ^1^ Faculty of Biomedical Sciences University of Leeds Leeds LS2 9JT UK; ^2^ Leeds Institute of Cardiovascular and Metabolic Medicine University of Leeds Leeds UK

**Keywords:** HFrEF, DM, Muscle wasting, Proteolysis, Anabolic, Insulin

## Abstract

Two highly prevalent and growing global diseases impacted by skeletal muscle atrophy are chronic heart failure (HF) and type 2 diabetes mellitus (DM). The presence of either condition increases the likelihood of developing the other, with recent studies revealing a large and relatively poorly characterized clinical population of patients with coexistent HF and DM (HFDM). HFDM results in worse symptoms and poorer clinical outcomes compared with DM or HF alone, and cardiovascular‐focused disease‐modifying agents have proven less effective in HFDM indicating a key role of the periphery. This review combines current clinical knowledge and basic biological mechanisms to address the critical emergence of skeletal muscle atrophy in patients with HFDM as a key driver of symptoms. We discuss how the degree of skeletal muscle wasting in patients with HFDM is likely underpinned by a variety of mechanisms that include mitochondrial dysfunction, insulin resistance, inflammation, and lipotoxicity. Given many atrophic triggers (e.g. ubiquitin proteasome/autophagy/calpain activity and supressed IGF1‐Akt‐mTORC1 signalling) are linked to increased production of reactive oxygen species, we speculate that a higher pro‐oxidative state in HFDM could be a unifying mechanism that promotes accelerated fibre atrophy. Overall, our proposal is that patients with HFDM represent a unique clinical population, prompting a review of treatment strategies including further focus on elucidating potential mechanisms and therapeutic targets of muscle atrophy in these distinct patients.

## Introduction

Skeletal muscle is one of the largest organs in the human body, accounting for approximately 40% of total body mass and acting as a major site for both protein storage and glucose disposal.^1^ Wasting of skeletal muscle, referred to as atrophy, is characteristic of several catabolic conditions, including aging (i.e. sarcopenia), starvation, and immobilization, but also occurs as a consequence of chronic disease.^2^, ^3^ Cachexia is closely linked to muscle atrophy and is defined as a complex multifactorial metabolic syndrome, which is associated with a significant reduction in body mass underpinned by skeletal muscle loss (with or without fat mass loss) and not fully reversible with nutritional aids.^4^ On the other hand, sarcopenia is the slow and progressive loss of muscle mass without any underlying disease, associated with advanced age.^4^ Collectively, a loss of muscle mass leads to a decline in functional mobility thereby contributing to poor quality of life and worse survival.^5^

Two highly prevalent chronic diseases implicated in and impacted by muscle atrophy include chronic heart failure (HF)^4^ and type 2 diabetes mellitus (DM).^6^ HF is a clinical syndrome resulting from structural or functional abnormality of the heart characterized by symptoms of breathlessness, exercise intolerance, and fatigue. The presence of DM in patients with HF, which in this review we term HFDM, has a synergistic adverse effect on both symptoms and prognosis.^7^ Exercise intolerance in HF has traditionally been understood in terms of a haemodynamic model, in which poor perfusion of skeletal muscle due to left ventricular (LV) dysfunction results in the sensation of breathlessness, while increased LV filling pressures result in reduced pulmonary diffusion due to interstitial oedema. However, this model fails to explain evidence that first measures of LV dysfunction correlate poorly with symptoms: patients with severely impaired systolic dysfunction can be asymptomatic, while those with mild impairments can be very limited.^8^ Second, patients who recover their LV systolic function either through disease modifying pharmacotherapy,^9^ implantable cardiac electronic devices,^10^ or even heart transplantation^11^ continue to have marked impairments of exercise capacity compared with healthy individuals. And finally, although exercise training can result in dramatic improvements in exercise capacity, these interventions have little or no effect on cardiac function.^12^ That exercise limitation in HF does not relate to the degree of cardiac dysfunction suggest that a proportion of symptoms in patients with HF, and especially HFDM, is driven by peripheral impairments including skeletal muscle myopathy. However, there remains little in the literature detailing the impact of muscle atrophy and its relation to symptoms and clinical outcomes in patients who have both HF and DM.

This review combines current clinical knowledge and basic biological mechanisms to address the important and emerging issue of skeletal muscle atrophy in patients with HFDM. We aim to answer a number of specific questions in relation to patients with HFDM when compared with those with HF or DM alone, including (i) Is there a distinct clinical and skeletal muscle phenotype? (ii) To what degree is muscle atrophy exacerbated? (iii) Are distinct molecular mechanisms mediating muscle atrophy? and (iv) What knowledge gaps and future directions should be pursued? We will primarily discuss evidence related to humans in order to maintain clinical translation and highlight areas lacking clarity, while we will focus on patients with type 2 DM and HF with reduced ejection fraction because the clinical phenotyping of HF with reduced ejection fraction is somewhat more established than HF with preserved ejection fraction.

## Does heart failure with diabetes represent a distinct clinical population?

Both HF and DM are growing global health problems, with a prevalence of 26 million^13^ and 374 million,^14^ respectively. Importantly, the presence of either HF or DM increases the likelihood of developing the other, with around 30% of patients with HF developing DM,^15^, ^16^, ^17^ while around 15% of patients with DM developing HF.^18^, ^19^, ^20^ DM is not only a risk factor for the development of HF but is also associated with increased risk of progressive HF and cardiovascular deaths in these patients.^19^, ^20^, ^21^ On the other hand, the HF syndrome is associated with insulin resistance,^22^ while pharmacological therapies for HF targeting the renin–angiotensin–aldosterone system^23^ and LV assist devices^24^ improve glycaemic control in patients with HFDM. Recent studies have revealed a large, novel, and poorly characterized clinical population of patients with coexistent HF and DM (i.e. HFDM).^16^, ^25^, ^26^, ^27^, ^28^, ^29^, ^30^, ^31^ However, our clinical understanding remains limited due to it being unclear whether DM is causal or an associative comorbidity in HF.^32^ As such, determining whether HF or DM occurs first may be an important factor shaping clinical outcomes and the severity of subsequent peripheral maladaptations.

**Figure 1:**
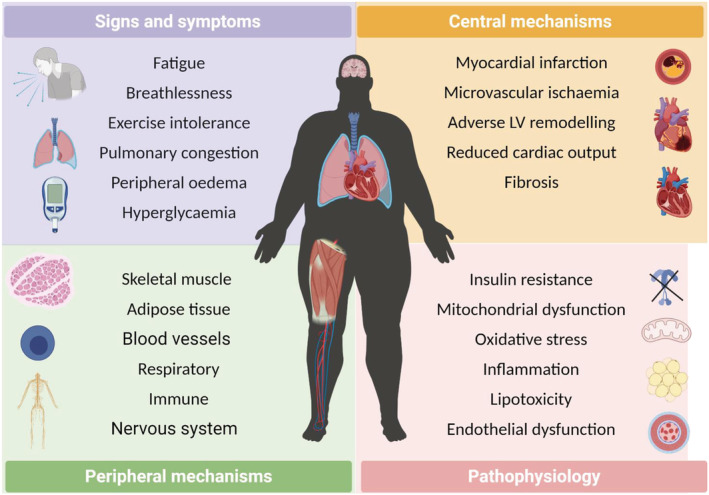
Systemic consequences of heart failure and diabetes, which includes signs and symptoms, central and peripheral mechanisms, alongside underlying biological pathophysiology with a specific focus on potential triggers of exacerbated skeletal muscle atrophy in patients suffering both conditions.

In particular, the development of HF and DM together is associated with many systemic organ abnormalities that extend beyond the heart, including activation of the renin–angiotensin–aldosterone and autonomic nervous systems, an elevated immune and inflammatory response, macrovascular and microvascular dysfunction, disturbed whole‐body energy metabolism, pulmonary impairments, and maladaptation of skeletal muscle and adipose tissue (*Figure* *1*). It should come as no surprise, therefore, that patients with HFDM have both worse outcomes and symptoms compared with those with HF or DM.^16^, ^33^ For example, peak pulmonary oxygen uptake (V̇O_2peak_), the gold‐standard measure of exercise intolerance and a strong predictor of mortality,^27^ is lower by around 15–20% in HFDM vs. HF or DM despite a similar degree of cardiac dysfunction.^27^, ^34^, ^35^ The most commonly used scoring system for symptoms, the New York Heart Association functional class, which divides patients into four subjective points on the basis of functional capacity reveals that patients with HFDM are on average more symptomatic and also have higher diuretic requirements compared with patients suffering HF without DM.^36^ Collectively, the poor relationship between LV systolic dysfunction and symptoms in HF with and without DM suggest an important role of peripheral mechanisms.^37^ The latter is reinforced by current data showing that patients with HFDM have not responded favourably to various cardiac‐orientated pharmacological treatments that have otherwise proven effective in patients with HF or DM,^38^, ^39^, ^40^, ^41^, ^42^, ^43^ although sodium glucose co‐transporter 2 (SGLT2) inhibitors may be one recent exception in which there is no interaction with the presence or absence of DM.^44^, ^45^ Furthermore, recent molecular network blood profiling has revealed a distinct phenotype between HF with and without DM in terms of inflammation, fibrosis, and neutrophil degranulation.^30^, ^31^ Taken together, patients with HFDM display an adverse clinical phenotype and this may be underpinned, at least in part, by more severe peripheral abnormalities originating in the skeletal muscles. Indeed, decreased muscle strength observed in HF or DM^46^ is more pronounced in patients who have both.^29^ As such, skeletal muscle could represent an important therapeutic target in patients with HFDM that otherwise respond poorly to current cardiometabolic orientated medications.

## Is skeletal muscle wasting exacerbated in patients with heart failure and diabetes?

Skeletal muscle atrophy is common in patients with HF and is independently associated with increased mortality.^5^ There is considerable overlap between muscle atrophy and cachexia in HF,^4^ which occurs in 20–40%^47^, ^48^ and 10–15%^49^ of patients, respectively. DM is similarly associated with increased muscle atrophy compared with controls with a prevalence of approximately 15%, which is linked to a poorer prognosis.^50^ Muscle loss can be measured *in vivo* through various techniques including non‐invasive computerized tomography and dual‐energy x‐ray absorptiometry, while direct invasive biopsies provide an opportunity to assess fibre cross‐sectional area (FCSA) and phenotype. In humans, it is generally agreed that three skeletal muscle fibre isoforms exist including types I, IIA, and IIX, progressing from small, fatigue resistant with high mitochondrial content and vascular supply towards the larger and higher force generating but more fatigable fibres, respectively.^51^

Several studies have shown that patients with HF or DM have reduced FCSA, and in particular that of IIA/X fibre types.^52^ There is also a shift from predominantly type I muscle fibre proportion towards more type II fibres in HF.^25^, ^53^ However, in contrast to HF where type IIX proportion is the greatest, type IIA fibres show a higher proportion over types I and IIX in DM.^25^ As predicted, reduced muscle mass is coupled to loss of muscle strength in patients with HF or DM,^5^, ^54^, ^55^ which provides the close link between maintaining muscle mass and functional status. While data for the muscle phenotype in HFDM remains scarce, initial evidence has shown muscle atrophy and functional impairments are more severe compared with HF or DM alone: patients with HFDM have an increased fibre atrophy in the pectoralis major (30% and 25% reduction in total FCSA, respectively),^25^ which is paralleled by impaired muscle strength (i.e. ~15% decrease in knee extensor/flexor measurements alongside a 10% decrease in handgrip strength).^29^ In general, patients with HF or DM show a type II fibre‐specific atrophy independent of type I^25^ and recent data in patients with HFDM show a similar trend: type IIA FCSA was reduced by approximately 40% and type IIX by approximately 50% compared with HF but to a lesser degree compared with patients with DM.^25^ This finding is important, as one may speculate both type I *and* II fibres would be impacted if patients with HFDM suffer a unique myopathy. These findings also highlight that the degree of type II fibre atrophy in HFDM was substantially higher compared with that in HF patients but not patients with DM,^25^ suggesting that DM may be the principle pathology inducing a greater atrophic phenotype in HFDM, which was recently indicated.^56^ Recent data using dual‐energy x‐ray absorptiometry have also confirmed that appendicular skeletal muscle mass index was reduced by almost 10% in large cohort of 70 patients with HFDM vs. 115 patients with HF.^56^ Given that muscle strength is also influenced by other factors such as lipid infiltration, patients with HFDM show higher body fat measures by 10–25% compared with those patients with HF,^57^ which may elevate intramyofibre lipid infiltration to further limit functional capacity. Overall, current data support the notion that patients with HFDM have altered body composition, with increased body fat and reduced skeletal muscle mass alongside impaired functional strength when compared with HF or DM populations.^25^, ^29^, ^57^ Limited evidence is available to support the existence of a unique myopathy in HFDM; however, current conclusions are based on only a limited number of studies, and more data from larger sample sizes are warranted.

## What molecular mechanisms control muscle mass in heart failure and diabetes?

**Figure 2:**
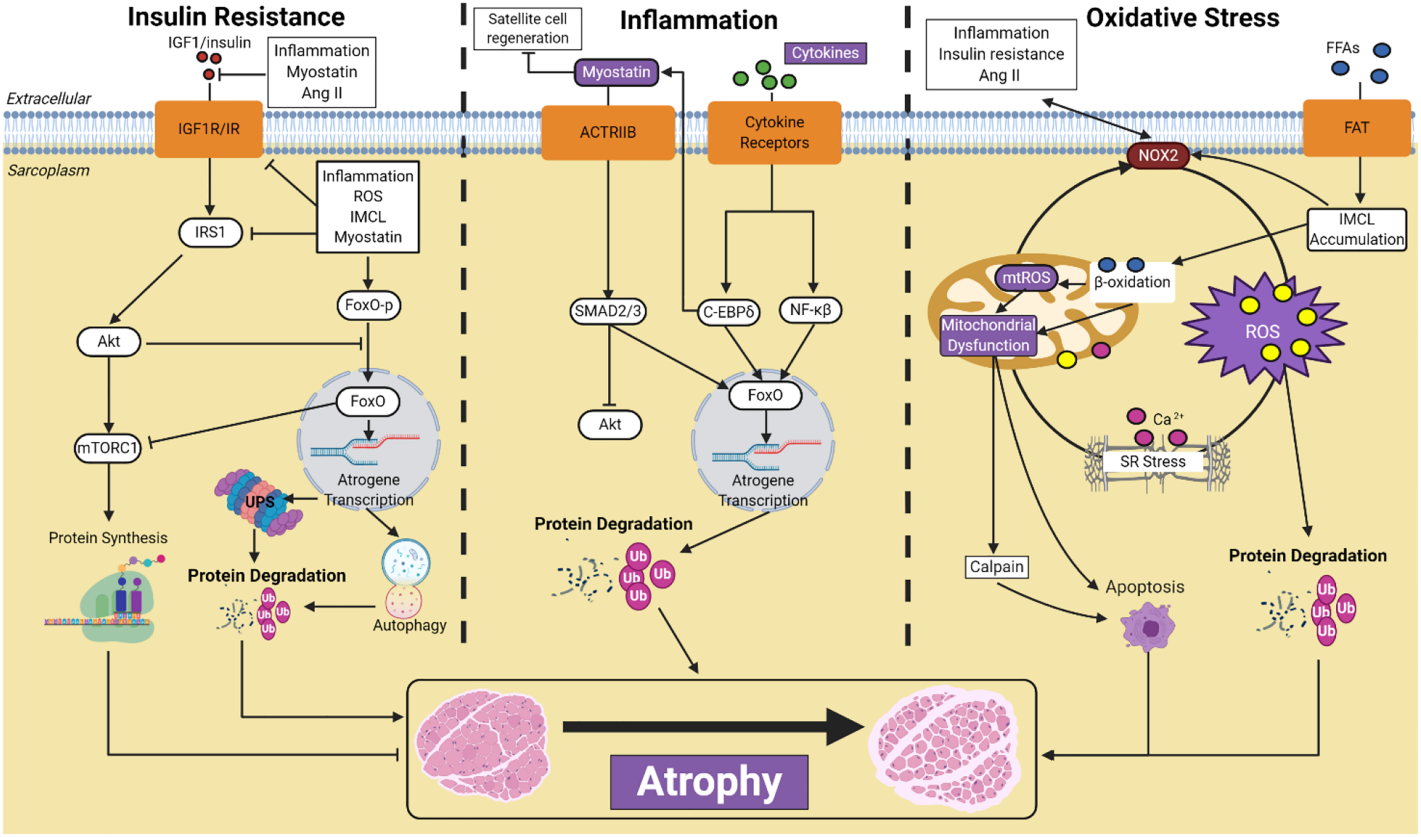
Hypothesis of the key molecular pathways involved in exacerbating skeletal muscle atrophy in patients with heart failure and type 2 diabetes mellitus (HFDM). The first column *Insulin Resistance* shows simplified mechanisms governing atrophy including regulation of protein degradation and protein synthesis in relation to suppression of IGF1/Akt/mTORC1 signalling and up‐regulation of atrogene transcription. Other major upstream mechanisms thought to be involved include *Inflammation* and *Oxidative stress*, with the latter closely linked with mitochondrial dysfunction as a control mechanism in many of the atrophic pathways. Labels in *purple* indicate evidence available to show significantly altered in patients with HFDM compared with those with DM or HF. See text for full details and defined abbreviations.

Given that current evidence indicates fibre atrophy is higher in patients with HFDM compared with those with HF or DM by around approximately 25%, the next question one naturally poses is what mechanisms are mediating this response? In general, muscle mass is controlled by the complex balance between rates of protein synthesis and rates of protein degradation. The molecular signalling pathways that interact to control protein synthesis/degradation generally include the insulin‐like growth factor 1 (IGF1)/protein kinase B (Akt)/mammalian target of rapamycin (mTOR)‐forkhead box protein O (FoxO), TGF‐β/myostatin/bone morphogenetic protein (BMP), nuclear factor kappa‐light‐chain‐enhancer of activated B cells (NF‐κB), and glucocorticoid^58^ (*Figure* *2*), which are flexible to modulation.^3^ These signalling pathways regulate the fate of many proteins, which include degradation by one of the four major proteolytic systems in the cell: ubiquitin‐proteasome, autophagy‐lysosome, calpain, and caspase.^58^ Typically, in atrophic conditions, evidence exists to support that proteolysis is elevated while protein synthesis is blunted; however, there is ongoing debate as to whether elevated proteolysis or supressed protein synthesis is the primary mechanism responsible for atrophy in catabolic conditions, which seems to be highly dependent on the clinical condition and experimental model employed.^52^, ^59^, ^60^ For example, protein synthesis and degradation are three‐fold and two‐fold higher in rodents compared with those in humans under catabolic conditions, respectively.^59^ Given such evidence, here we focus our attention on human data to provide greater relevance to patients and aid clinical understanding.

### Role of proteolysis

The role of the key proteolytic pathways in skeletal muscle from patients with HF, DM, and HFDM remains poorly defined, with a limited number of comprehensive studies addressing this issue. The ubiquitin proteasome system is recognized as the most important proteolytic pathway to mediate muscle atrophy,^1^ with a major rate‐limiting step in this process being attachment of ubiquitin to target proteins via muscle‐specific E3 ubiquitin ligases termed atrogenes, which include MAFbx and MuRF1 (*Figure* *2*).^61^, ^62^ MuRF‐1 mRNA and protein expression have been reported to be elevated in *vastus lateralis* biopsies of patients with HF compared with age‐matched controls,^63^ which is also the case for patients with DM.^64^ In contrast, MAFbx generally seems to remain unchanged between HF or DM groups when compared with controls,^63^, ^64^, ^65^ suggesting that MuRF1 may play a more important role mediating atrophy in HF and DM. However, there remains a lack of consistency in the literature, with MuRF‐1 and MAFbx mRNA expression reported to be unchanged in HF compared with controls,^63^, ^65^ while ubiquitin mRNA expression has been found to be significantly decreased in DM patients^66^ but increased in HF patients.^64^ In relation to HFDM, almost no studies have measured levels of MuRF1 or MAFbx. One study reported MAFbx and MuRF1 mRNA levels were unchanged between controls and patients with DM, HF, or HFDM groups^64^; however, this study was underpowered with no information on fibre atrophy.

The machinery involved in protein degradation are largely controlled by a subset of highly regulated transcriptional factors that can induce proteolytic activity, with the FoxO and NF‐κB transcription factors central^58^ (*Figure* *2*). FoxO1 and FoxO3 mRNA expression has been shown to be up‐regulated in patients with HF compared with controls, but unchanged between DM and HFDM.^64^ In relation to NF‐κB, it seems likely this would be elevated in patients with HFDM given increased levels reported in both patients with HF or DM.^67^, ^68^ The other major proteolytic system involved in wasting, which can also be regulated by FoxO transcription, is the autophagy‐lysosomal pathway (*Figure* *2*). This pathway targets damaged organelles for removal, such as the mitochondria, by forming autophagosomes, which subsequently fuse and undergo lysosomal degradation.^58^ In DM, the expression of autophagy‐related genes including *ULK1*, microtubule‐associated protein 1 light chain 3 (*LC3*), and *p62* have been observed to be either unchanged^69^ or down‐regulated compared with those in healthy controls.^70^ In contrast, autophagy‐related expression in muscle biopsies from patients with HF is limited, with one study showing cathepsin L (a key protease involved in lysosomal degradation) was not different in HF compared with controls.^63^ No data are currently available for patients with HFDM for the other proteolytic pathways of calpain and caspase‐3. Overall, therefore, more data are required to clarify the contribution of key proteolytic pathways and transcription factors and their link to fibre atrophy in patients with HFDM alongside additional whole‐body measures of protein metabolism for additional insight.^71^

### Role of protein synthesis

One of the main pathways controlling protein translation and muscle growth is the insulin‐IGF1/Akt/mTORC1 signalling axis (*Figure* *2*). Importantly, there is close crosstalk between anabolic and catabolic signalling pathways, for example, Akt can suppress atrophy *via* inhibiting FoxO signalling.^72^ In both HF and DM, several proteins within the anabolic pathway are known to be decreased or inhibited. Although not always consistent,^64^ IGF‐1 mRNA expression has been found to be significantly reduced (>50%) in skeletal muscle of HF patients compared with those in healthy controls, which was verified by protein levels.^73^ Also IGF binding protein 5 (IGFBP5) mRNA expression is significantly increased in both HF^64^ and DM^66^ patients, indicating supressed anabolic signalling as IGFBP5 can inhibit IGF‐1 signalling and thus mTORC1 activation,^74^ while lower levels of serum IGFBP3 alongside evidence of growth hormone resistance have been reported in HF patients.^75^ Further support comes from measures of insulin receptor substrate 1 (IRS1), which links insulin/IGF signalling to PI3K/Akt, whereby mRNA expression for IRS1 was reported to be decreased by almost three‐fold in patients with DM compared with those in healthy controls highlighting the major role insulin resistance may play.^66^ Despite total Akt and mTORC1 protein expression remaining unchanged in HF or DM,^70^, ^76^, ^77^ the phosphorylated and thus activated form of these kinases was reduced in both diseases, suggesting that protein synthesis is likely diminished.^70^, ^76^, ^77^ However, in regard to HFDM, there remains limited data to support whether anabolic signalling is suppressed and more studies are warranted.

## What upstream triggers could exacerbate muscle atrophy in heart failure and diabetes?

The anabolic and proteolytic systems controlling muscle mass are tightly governed by upstream factors related to hormonal/cytokine, metabolic/nutrient, mechanical load, and neural activity, which helps to explain why conditions such as starvation, exercise, immobilization, and disease‐related conditions induce rapid fluctuations in muscle mass. There are several mechanisms that could contribute towards increased skeletal muscle atrophy, by acting to elevate protein degradation and blunt protein synthesis, in patients with HFDM compared with those with HF or DM alone as summarize in *Figure* *2*. These likely include insulin resistance/hyperglycaemia, inflammation, oxidative stress, mitochondrial dysfunction, lipotoxicity, hormonal resistance, disuse, and vascular dysfunction.

### Insulin resistance and hyperglycaemia

Insulin is a hormone with multiple effects and plays a major role in the regulation of muscle metabolism (i.e. glucose uptake), but also in controlling muscle mass via the close association with IGF1 via PI3K/Akt/mTORC1 signalling. Contracting skeletal muscle is a major deposition site for glucose, which is facilitated by insulin‐stimulated glucose transporter type 4 (GLUT4) uptake. Impairments to the insulin‐GLUT4 pathway predisposes towards hyperglycaemia and GLUT4 levels are reduced in both patients with DM^78^ and HF.^79^ Insulin resistance is considered one major defect in the pathology of DM in humans.^80^ Insulin sensitivity is also known to be lower in HF patients without DM and has been linked to reductions in both muscle quantity and function.^79^, ^81^, ^82^, ^83^ IGF‐1 and IRS1 gene expression are significantly decreased in DM and HF patients,^66^, ^73^ with impaired phosphorylation of IRS1 in DM reported to contribute to lowering insulin sensitivity.^78^ IRS1 signalling can also be impaired following targeted degradation by the E3 ligases mitsugumin 53 (MG53) and F‐box protein 40 (FBXO40).^84^, ^85^ Altered phosphorylation and/or ubiquitination of IRS1 could exacerbate muscle atrophy by reducing Akt activation, which could lead to increased degradation via FoxO‐dependent atrogene transcription while simultaneously reducing protein synthesis via inhibition of downstream mTORC1 signalling. While no data are available in patients with HFDM to address this issue, greater insulin resistance is expected to be a major upstream mechanism mediating fibre atrophy in these patients (*Figure* *2*). Impaired insulin sensitivity can also lead to hyperglycaemia, and while direct data from humans are missing, animal models of DM have shown a causal link to fibre atrophy via the WWP1/KLF15 pathway^86^ as well as contractile dysfunction,^86^, ^87^ ROS,^88^ and endothelial dysfunction.^89^ Further evidence indicates that a reduction in muscle contractions *per se* can also drive the development of insulin resistance and in combination with a systemic inflammation, can impair insulin insensitivity to further exacerbate muscle wasting by reducing protein synthesis and elevating protein degradation.^52^ This highlights the importance of implementing exercise training in patients with HFDM as a treatment for muscle atrophy.^2^

### Inflammation and circulating factors

A pro‐inflammatory state is common in both patients with HF or DM, which is characterized by chronic increases in levels of cytokines that include TNF‐α, interleukin 1 and 6 (IL1, IL‐6).^6^, ^90^ TNF‐α can activate the key transcription factor NF‐κΒ, and this translocates from cytoplasm to nucleus, mediating increased protein degradation in a MuRF‐1 dependent manner, while it can also induce insulin resistance via increased serine phosphorylation of IRS1^6^, ^91^ and increase ROS production (*Figure* *2*). IL‐6 activates the Janus kinase/signal transducer and activator of transcription proteins (JAK/STAT) pathway, which is also implicated in promoting protein degradation and insulin resistance in patients.^6^ Circulating and skeletal muscle levels of these key pro‐inflammatory cytokines have been reported to be elevated in patients with HF^67^, ^68^, ^92^ and DM,^68^, ^93^ which strongly correlate with mortality.^92^ In relation to HFDM, our knowledge is limited, but recent network analyses of blood samples in two separate studies have identified inflammation as being an up‐regulated pathway in patients with HFDM compared with those with HF^30^, ^31^ but muscle atrophy was not measured in this study. Overall, therefore, it seems that inflammation is higher in patients with HFDM vs. HF or DM, which could act as a major upstream mechanism accelerating fibre atrophy. Myostatin is a member of the transforming growth factor beta (TGF‐β) superfamily and a negative regulator of muscle mass, which acts via phosphorylated Smad2/3 to inhibit Akt signalling as well as inhibiting satellite cell function (*Figure* *2*). Circulating myostatin is elevated in patients with HF^94^ or DM,^95^ while mRNA expression in skeletal muscle is reported to be either elevated or unchanged.^64^, ^95^ In relation to HFDM, some evidence indicates that myostatin expression is elevated compared with healthy controls in skeletal muscle.^26^ However, myostatin expression was not correlated to fibre atrophy or downstream targets such as SMAD2/3 or Akt, thus limiting interpretation. Angiotensin II (Ang II) is a hormone that functions to both promote protein degradation and inhibit protein synthesis (*Figure* *2*). Ang II impairs Akt and mTORC1 signalling by inhibiting the IGF1 signalling axis while simultaneously promoting atrogene transcription,^96^ alongside inducing ROS production as well as insulin resistance via inhibition of IRS1 via protein kinase C (PKC) activation.^97^ Circulating levels of Ang II are increased in patients with HF^98^; however, a significant decrease has been reported in patients with DM compared with those in controls.^99^ While no direct data are available for HFDM, recent data have shown that plasma renin activity was higher and negatively correlated to muscle mass (i.e. appendicular skeletal muscle mass index) in patients with HFDM but not HF, suggesting a role for the renin–angiotensin system^56^ that could act downstream via ROS‐dependent signalling to induce wasting (*Figure* *2*).

### Mitochondrial dysfunction

While the mitochondria were traditionally viewed as being simply energetic organelles, recent evidence now show they play a key signalling role, which in turn, may control muscle wasting (*Figure* *2*). It is well established that both patients with HF or DM develop mitochondrial dysfunction, which include for example a lower respiratory function and content.^25^, ^100^, ^101^, ^102^ In patients with HFDM, mitochondrial content has been estimated to be lower by 25%, while mitochondrial dysfunction assessed *in situ* using high‐resolution respirometry showed impairments specific to complex I both absolute and when normalized to mitochondrial content, as assessed in the pectoralis major when compared with patients with HF or DM.^25^ This finding is important, as it suggests a complex I‐specific impairment related to both quality and quantity in patients with HFDM but not HF or DM, and importantly, this was correlated to whole‐body measures of exercise intolerance.^25^ Further experiments revealed that impairments at the transcriptional level are present, with gene expression of the main subunit of complex I (NADH ubiquinone oxidoreductase core subunit S1; NDUFS1) lower in HFDM compared with other groups.^25^ Further support for intrinsic mitochondrial myopathy in patients with HFDM comes from evidence that measures of mitochondrial coupling (i.e. respiratory control ratio) was similar to controls in both HF and DM, but significantly lower by almost one third in HFDM.^25^ Such evidence provides support that mitochondrial dysfunction is exacerbated in patients with HFDM compared with those with HF or DM, which may be a unique manifestation and key link to the degree of fibre atrophy reported. Interestingly, supplementation of 100 mg (−)‐epicatechin per day for 3 months (a type of natural phenol and antioxidant) has shown beneficial effects on mitochondrial function in patients with HFDM, including on mitochondrial volume and size.^103^ This was attributed to improvements in the expression of key regulators of oxidative metabolism including sirtuin 1 (SIRT1), PGC‐1α, transcription factor A mitochondrial (TFAM), and neuronal NO synthase (nNOS),^104^ but also to epicatechin's anti‐oxidative properties as measures of both glutathione, catalase, and SOD2 in HFDM were normalized towards control values.^26^ While these studies should be interpreted with caution due to the low sample size and lack of adequate HF, DM, or healthy age‐matched controls throughout, they offer important insight that HFDM may be linked to mitochondrial dysfunction.

### Oxidative stress

Mitochondrial impairments have been associated with increased ROS production in skeletal muscle of both patients with HF or DM. ROS have important roles in cell signalling and homeostasis; however, significant elevations in ROS can lead to oxidative stress and have serious adverse effects on myofibre stability.^105^ ROS production can also be stimulated by insulin resistance, hyperglycaemia, lipotoxicity, inflammation, endothelial dysfunction, and mitochondrial dysfunction, all promoting potential skeletal muscle atrophy (*Figure* *2*). The main sources of ROS in skeletal muscle are the mitochondria, nicotinamide adenine dinucleotide phosphate (NADPH) oxidase, xanthine oxidase, and uncoupled nitric oxide synthase (NOS),^106^, ^107^, ^108^ and evidence exists to suggest that ROS are elevated in patients with HFDM. For example, mitochondrial ROS production in permeabilized myofibres of the pectoralis major were shown to be the highest in patients with HFDM during complex I respiration compared with HF, DM, or control groups.^25^ Specifically, H_2_O_2_ production was greater in HFDM compared to DM or HF, which was accompanied by a significant decrease in gene expression of HFDM muscle of the mitochondrial‐specific antioxidant superoxide dismutase 2 (SOD2).^25^ Other studies have found that HFDM patients show reduced skeletal muscle glutathione levels and this is accompanied by a significant increase of carbonylation and nitrotyrosine residues (markers of oxidative damage) with increased acetylation and lower content of SOD2.^26^ A number of other studies have also revealed significantly higher levels of plasma oxidative stress markers and reduced levels of antioxidants in HFDM, in particular glutathione.^109^, ^110^, ^111^

Based on previous cell culture and animal experiments, increased ROS production promotes fibre atrophy via numerous signalling pathways (*Figure* *2*). ROS can increase pro‐inflammatory cytokine expression such as TNF‐α, thus activating NF‐κΒ signalling and atrogene transcription, while further evidence have shown that ROS can increase catabolic signalling via FoxO‐proteasome activation alongside supressing anabolic Akt‐mTORC1 signalling.^58^ ROS also damage proteins involved in the electron transport chain, which can further increase mitochondrial ROS production,^112^ in addition to damaging mitochondrial DNA (mtDNA) that can result in mitochondrial dysfunction.^106^ Increasing evidence suggests that superoxide produced by complex I is released into the matrix where its accumulation, together with the decrease in SOD2 activity, can result in significant mtDNA damage.^113^, ^114^ ROS can also promote calcium leak from the sarcoplasmic reticulum (SR), which can accumulate in the mitochondria to activate Bcl‐2‐associated X protein (Bax) and subsequent release of cytochrome C via the mitochondrial transition pore. This process can up‐regulate proteolytic pathways related to caspase/apoptosis and calpain signalling.^115^ Finally, ROS can also promote endothelial dysfunction, by reducing levels of nitric oxide (NO) and thus impairing vascular function and decreasing blood flow.^116^ Despite the limited number of studies, early data suggest that alterations in redox homeostasis may play a fundamental role in muscle wasting in patients with HFDM.

### Lipotoxicity

Abnormal substrate metabolism in skeletal muscle is a common manifestation in cardiometabolic disorders and this may lead to lipotoxicity, which has been closely linked to muscle atrophy.^117^ Lipotoxicity results from the accumulation of intramyocellular lipids (IMCLs) and increased concentration of associated species termed diacylglycerols (DAGs) and ceramides, which can be detrimental to skeletal muscle homeostasis.^118^ DAGs are especially detrimental as they can activate PKC, which can impair IRS1 signalling to impinge insulin sensitivity.^46^ IMCL accumulation has also been linked to increased mitochondrial dysfunction, ROS production, pro‐inflammatory cytokines, and ER stress, which can all contribute to skeletal muscle atrophy.^115^ DAG production is increased in skeletal muscle of patients with DM and is positively correlated to increases in PKC,^119^ while similar data have been reported in patients with HF.^120^ Increased accumulation of IMCL also predisposes towards mitochondrial lipid oxidation, thus overloading the electron transport chain and thereby increasing ROS generation.^115^ Currently, little data exist as to whether IMCL are elevated in patients with HFDM, but given current data in HF or DM, it remains one likely mechanism that would exacerbate wasting.

### Vascular dysfunction

The microvasculature of the skeletal muscle plays an essential role in transporting blood containing oxygen, amino acids, and nutrients to the muscle from the vascular system. There remains wide variability in findings as to whether skeletal muscle vascular measures are changed in patients with either HF or DM, with data showing an increase, decreased, or no change.^116^, ^121^, ^122^ In relation to patients with HFDM, pectoralis major capillary‐to‐fibre ratio was unchanged compared with those with HF, DM, or controls, and as such, the capillary density increased in HFDM because of the fibre atrophy.^25^ These data would argue that skeletal muscle capillarization is not contributing to skeletal muscle atrophy in HFDM. However, this does not rule out blood flow being reduced in patients with HFDM to impact muscle function. Endothelial dysfunction is characterized by impaired vasodilation, due to a reduction in NO and/or an increase in vasoconstricting factors such as endothelin 1 (ET‐1), which can decrease blood flow to subsequently limit oxygen transport and nutrient supply to skeletal muscle.^123^ Impaired oxygen transport can also induce hypoxia that has potential to induce apoptosis and increase ROS production in muscle. At present, glucose uptake and blood flow have been shown to be significantly decreased in HF or DM patients compared with controls, and even further in HFDM, which indicates an additive impairment.^28^ It seems, therefore, that patients with HFDM have functional limitations to skeletal muscle blood flow rather than structural impairments, which may contribute towards fibre atrophy.

## Clinical translation and future perspectives

In the past two decades, we have made great strides in improving outcomes for patients living with HF, as well as developing new agents to treat DM. However, increased longevity has not been mirrored by improvements in symptoms, and every year more patients are living with symptoms of HF. Moving forwards, future studies are required from independent groups confirming the degree of skeletal muscle atrophy and phenotypical alterations in patients with HFDM. To date, few studies have investigated direct mechanisms contributing to skeletal muscle atrophy in HF or DM, and this is especially true for patients with HFDM, where more evidence is required for cause or consequence. As such, interventional studies are required to identify mechanisms involved in skeletal muscle remodelling and atrophy in patients with HFDM and whether they are common in DM or/and HF or indeed representative of a unique clinical phenotype (*Figure* *2*). While differences between upper and lower limb muscles have been assessed in regard to strength,^29^ the diaphragm represents another muscle worthy of investigation in HFDM, particularly as studies have shown that diaphragm dysfunction occurs in HF patients and is closely related to prognosis.^124^ Disuse atrophy can also contribute to muscle wasting in various diseases, and studies have identified that patients with HFDM compared with those with HF are less likely to be referred for, and participate in, cardiac rehabilitation.^125^ As such, the role of physical inactivity should be investigated as one potential factor that may exacerbate muscle atrophy in patients with HFDM, while improving referral and participation should also be encouraged given its benefits on muscle atrophy.^2^ One major issue to address is whether patients with HFDM represent a unique clinical phenotype or an additive result of DM and HF pathologies, both at the clinical and muscle tissue and at the molecular level. At present, there are not enough data to accept or refute these notions, with more extensive data and interventions required to answer this question, which is further limited by the lack of adequate pre‐clinical experimental models of HFDM that closely reflect the patient and skeletal muscle phenotype.

## Conclusions

The prevalence of HFDM is increasing globally, and these patients have worse symptoms and poorer survival compared to patients with HF or DM alone, while traditional pharmacological treatments show limited benefits. Despite much research performed within the HF or DM conditions, limited evidence has been collected regarding the skeletal muscle phenotype in patients with HFDM. However, initial data indicate greater skeletal muscle dysfunction and atrophy in patients with HFDM, which may be an important factor causing worse symptoms and poorer clinical outcomes. Early evidence indicates that potential mechanisms of atrophy in HFDM could involve mitochondrial dysfunction, insulin resistance, inflammation, and lipotoxicity (*Figure* *2*). Many of the atrophic pathways and mechanisms discussed in this review are linked to increased ROS production (e.g. activation of protein degradation, blunted protein synthesis, mitochondrial dysfunction, inflammation, apoptosis, lipotoxicity, calcium leak, and endothelial dysfunction). As such, we speculate that a higher pro‐oxidative state in patients with HFDM could be one unifying mechanism mediating the exacerbated fibre atrophy in this clinical population. Future studies are clearly required to clarify these questions, and it should be seen as an urgent medical priority given that this patient population is expanding despite limited therapeutic treatments.

## Conflict of interest

N.W., S.S., M.S., L.R., and T.S.B. declare that they have no conflict of interest. K.K.W has received speakers' fees and honoraria from Medtronic, Cardiac Dimensions, Novartis, Abbott, BMS, Pfizer, and Bayer and has received unconditional research grants from Medtronic.

## Funding

S.S. is supported by a British Heart Foundation Scholarship. L.D.R. is supported by the Diabetes UK RD Lawrence Fellowship (16/0005382). T.S.B. is supported by MRC UK (MR/S025472/1) and Heart Research UK (TRP 16/19).
